# Supplementary material for: The genome sequence of the Crepuscular Burnet, Zygaena carniolica (Scopoli, 1763) (Lepidoptera: Zygaenidae)
Source: Wellcome Open Res. 2025 Jul 28;10:374. [Version 1] doi: 10.12688/wellcomeopenres.24616.1 (PMC12627941; doi:10.12688/wellcomeopenres.24616.1)
Supplement: Peer review history

# Reviewer report: Reviewer response for version 1

**DOI:** 10.21956/wellcomeopenres.27126.r139546

**Published:** 2026-01-05

**Authors:** Luana Bataglia

**Affiliations:** 1 Universita Cattolica del Sacro Cuore - Campus di Piacenza e Cremona (Ringgold ID: 96985), Piacenza, Emilia-Romagna, Italy

The article reports the sequencing, at the chromosome level, of the genome of the Lepidoptera species *Zygaena carniolica*. 

 The article is sufficient as a data note, but I suggest a minimum of biological interpretation of the technical data provided. This will proportionate a gain in quality and visibility for the article.

 Some points to be considered are:

 1) The mitochondrial genome data were poorly reported. The only information is about the length of 15.56 kilobases, reported in the abstract. But this information is missing in the results section. 

 2) Although it is written that the gene annotation is still to be done, I strongly suggest that the authors complete it as soon as possible and include this information in the article.

 3) The authors should include small discussions as:

 - Do the length of the nuclear and mitochondrial genomes match the expected size and resemble those of close species?

 -The same kind of discussion should be done with the annotation data. Do the number of predicted genes and proteins match the expected number and resemble those of close species?

 - Some groups of genes presented a significant increase or decrease in the species?

Are sufficient details of methods and materials provided to allow replication by others?

Yes

Is the rationale for creating the dataset(s) clearly described?

Yes

Are the datasets clearly presented in a useable and accessible format?

Yes

Are the protocols appropriate and is the work technically sound?

Yes

Reviewer Expertise:

Molecular Biology, Genetics, Epigenetics, Entomology, Biotechnology.

I confirm that I have read this submission and believe that I have an appropriate level of expertise to confirm that it is of an acceptable scientific standard.

# Reviewer report: Reviewer response for version 1

**DOI:** 10.21956/wellcomeopenres.27126.r139549

**Published:** 2025-11-26

**Authors:** Matthew Merkin

**Affiliations:** 1 University of Liverpool, Liverpool, UK

The authors present a chromosome-level genome assembly for the Crepuscular Burnet moth (Zygaena carniolica), which was generated for Project Psyche. A female sample was collected in Switzerland and DNA was extracted then sequenced from its thorax and head to obtain HiFi and Hi-C reads respectively. The reads were then assembled, scaffolded and passed through several quality assessment pipelines to obtain the final sequence report. High quality score metrics, such as an EBP score of 6.C.Q64 and BUSCO completeness of 98% suggest that this assembly is suitable for use as a reference genome. 

 Minor comments:

 The introduction to this genome note is excellent at highlighting the interesting features of this species and previous genetic studies. However, there is a typo with "biovoltine" instead of "bivoltine" and the phrase "Flight time is variable" is ambiguous; it is unclear whether this refers to flight season (months of the year in which adults can be found) or time of day- the latter of which seems particularly relevant in this species due to its "crepuscular" title. Additionally, an extra sentence explaining what Project Psyche is and why this species was chosen in particular would be beneficial. 

 The consistent reference to the primary haplotype containing 31 chromosomes (e.g. abstract and Table 2) is very misleading since it implies that there are 30 autosomes and the sex chromosome. However,there are only 29 autosomes since the Z and W are both included in the primary assembly. As such, a reader skimming the start of the paper may assume that the haploid chromosome number is 31 instead of 30, which is particularly problematic since the introduction mentions that different populations can have 30 or 31 chromosomes (with this individual belonging to the former). Although this is briefly explained in a sentence below Table 2, I feel that it should be explicitly stated that n=30 in a prominent position e.g. abstract or end of the introduction.

 The number of significant figures that the mitochondrial genome is reported to is odd. In the text and Table 2, 15.56kb is used when 15561bp has a similar number of characters. Table 3 reports this as 0.02Mb, which does not seem useful- this is also not a chromosomal pseudomolecule, so the sequence should probably not be in the table.

 Although there is a commitment in the data availability section to annotate the genome later on, the lack of discussion about an annotation makes this genome note feel incomplete and its publication premature.

Are sufficient details of methods and materials provided to allow replication by others?

Yes

Is the rationale for creating the dataset(s) clearly described?

Yes

Are the datasets clearly presented in a useable and accessible format?

Yes

Are the protocols appropriate and is the work technically sound?

Yes

Reviewer Expertise:

Comparative genomics, genome assembly, genetics, entomology, ancient DNA, population genetics

I confirm that I have read this submission and believe that I have an appropriate level of expertise to confirm that it is of an acceptable scientific standard.

# Reviewer report: Reviewer response for version 1

**DOI:** 10.21956/wellcomeopenres.27126.r132211

**Published:** 2025-11-18

**Authors:** Annabel Whibley

**Affiliations:** 1 The University of Auckland, Auckland, New Zealand; 2 Grapevine Improvement, Bragato Research Institute, Lincoln, New Zealand

Espeland and colleagues describe the chromosome-level, haplotype-resolved genome assembly of the Crepuscular Burnet, *Zygaena carniolica,* obtained from a female sample collected in Switzerland *. *The natural history and population distrubtion/status of this species are well summarized. The standard DToL sequencing, assembly and reporting pipelines and templates have been implemented, with chromosomal painting included to identify Merian elements in this Lepidopteran assembly. The assembly quality is exceedingly good (formally 6.C.Q64), with contiguity, completeness, accuracy and contamination assessments underscoring this. The project metadata is comprehensive and public accessions are active.

 I would second the comment of Reviewer 1, that the experimental details of the HiC protocol could be enhanced by including the specific enzyme used for fragmentation.

Are sufficient details of methods and materials provided to allow replication by others?

Yes

Is the rationale for creating the dataset(s) clearly described?

Yes

Are the datasets clearly presented in a useable and accessible format?

Yes

Are the protocols appropriate and is the work technically sound?

Yes

Reviewer Expertise:

Genomics Bioinformatics

I confirm that I have read this submission and believe that I have an appropriate level of expertise to confirm that it is of an acceptable scientific standard.

# Reviewer report: Reviewer response for version 1

**DOI:** 10.21956/wellcomeopenres.27126.r132217

**Published:** 2025-09-25

**Authors:** Bin Zhang

**Affiliations:** 1 Qingdao Agricultural University, Shenzhen, China

This manuscript presents a high-quality, haplotype-resolved genome assembly of *Zygaena carniolica* (Crepuscular Burnet), a species of ecological and evolutionary interest in the Lepidoptera: Zygaenidae family. While the manuscript is strong overall, several minor revisions would improve its completeness and scientific impact:

 The methods describe using a “restriction enzyme master mix” for Hi-C sample preparation but do not specify the restriction enzyme(s) (e.g., *DpnII*, *HindIII*) or enzyme recognition site. This information is critical for evaluating Hi-C data quality (e.g., fragment size distribution, valid contact rate) and reproducing the scaffolding workflow.

Are sufficient details of methods and materials provided to allow replication by others?

Yes

Is the rationale for creating the dataset(s) clearly described?

Yes

Are the datasets clearly presented in a useable and accessible format?

Yes

Are the protocols appropriate and is the work technically sound?

Yes

Reviewer Expertise:

Entomology, Bioinformatics

I confirm that I have read this submission and believe that I have an appropriate level of expertise to confirm that it is of an acceptable scientific standard.
